# Effects of resistance-type and cycling-type high-intensity interval training on cardiorespiratory fitness, lower-body strength, and anaerobic fitness

**DOI:** 10.3389/fphys.2026.1847911

**Published:** 2026-06-18

**Authors:** Tianjiao Wang, Jun Mao, Shumin Bo

**Affiliations:** College of Kinesiology and Health, Capital University of Physical Education and Sports, Beijing, China

**Keywords:** active healthy men, cardiorespiratory fitness, cycling-type high-intensity interval training, lower-body strength, resistance-type high-intensity interval training

## Abstract

**Background:**

Comparative evidence regarding the effects of different high-intensity interval training (HIIT) modalities on cardiorespiratory fitness, lower-body strength, and anaerobic fitness in active young men remains limited. This study aimed to determine the effects of resistance-type high-intensity interval training (R-HIIT) and cycling-type high-intensity interval training (C-HIIT) on cardiorespiratory fitness, lower-body strength, and anaerobic fitness in active healthy young men (n=24).

**Methods:**

Twenty-four healthy active young men took part in either the C-HIIT (n=12) or R-HIIT (n=12) group. Maximum oxygen uptake (V̇O_2max_), 30s Wingate test, back squat 1RM, knee flexion and extension peak torque, and body composition were measured both before and after the 8-week intervention.

**Results:**

Significant main effects of time were observed for V̇O_2max_ (P<0.05), average power output (P<0.001), and peak power output of anaerobic fitness (P<0.001). For back squat 1RM, a significant time main effect (P<0.001) and time × group interactions effect (P<0.05) were found. *Post-hoc* analysis revealed that R-HIIT elicited a greater increase in 1RM than C-HIIT (P<0.05). For isokinetic knee strength at 60°·s^-1^, peak torque increased in both flexors and extensors (P<0.05). Notably, R-HIIT demonstrated a superior improvement in knee flexion peak torque compared with C-HIIT (P<0.05), while no significant between-group difference was found for knee extension. Fat mass (FM) reduced with C-HIIT (P<0.05) and R-HIIT (P<0.05).

**Conclusion:**

Eight weeks of C-HIIT and R-HIIT produced similar improvements in V̇O_2max_, anaerobic fitness, knee extension peak torque, and fat mass. Compared with C-HIIT, R-HIIT induced greater improvements in back squat 1RM and knee flexion peak torque.

**Clinical trial registration:**

www.chictr.org.cn, identifier ChiCTR2200056897.

## Introduction

Cardiorespiratory fitness (CRF) is a well-established, independent predictor of cardiovascular disease and all-cause mortality, recognized in scientific statements such as those from the American Heart Association as a fundamental component of health-related physical fitness (Lan et al., 2022; Raghuveer et al., 2020). Despite its critical importance, a concerning decline in CRF levels has been documented globally in recent decades, a trend notably observable among college students in China (Kodama, 2009). Concurrently, muscular strength is increasingly recognized as another vital and independent determinant of long-term health (Ortega et al., 2012). These observations underscore the necessity of identifying time-efficient exercise strategies that simultaneously improve both physiological domains.

High-intensity interval training (HIIT)—characterized by brief, intense efforts interspersed with recovery periods—has emerged as a potent and time-efficient modality for enhancing not only aerobic capacity but also anaerobic fitness (Hazell et al., 2010; Laursen, 2010). While its efficacy in improving metabolic and cardiorespiratory health is well documented, evidence regarding its capacity to induce meaningful gains in muscular strength remains comparatively limited (Nybo et al., 2010). Integrating dedicated strength training with HIIT could address this gap but would substantially increase total time commitment, reducing practicality (Astorino et al., 2012).

A promising and efficient alternative is resistance-based HIIT (R-HIIT), which incorporates resistance exercises (e.g., using free weights or bodyweight) into an interval framework (Mao et al., 2022; Wang et al., 2024). Unlike classical, cycling-based HIIT (C-HIIT), which primarily stimulates central cardiovascular adaptations, R-HIIT is theorized to provide a combined stimulus, potentially eliciting concurrent improvements in aerobic capacity, anaerobic performance, and muscular strength within a single session (Buckley et al., 2015; McRae et al., 2012). However, existing research on R-HIIT has largely focused on whole-body circuit regimens, leaving a gap in understanding the effects of protocols centered on multi-joint, high-repetition resistance exercises—such as barbell squats—on a comprehensive set of fitness outcomes, including CRF, lower-body strength, anaerobic power, and body composition.

Therefore, this study aimed to directly compare the effects of an 8-week C-HIIT program versus a back squat-based R-HIIT program on these key fitness parameters in a population of active college students. We hypothesized that both interventions would improve CRF, anaerobic fitness, and body composition, but that R-HIIT would yield superior enhancements in measurements of lower-body muscular strength.

## Methods

### Study design

**Figure 1:**
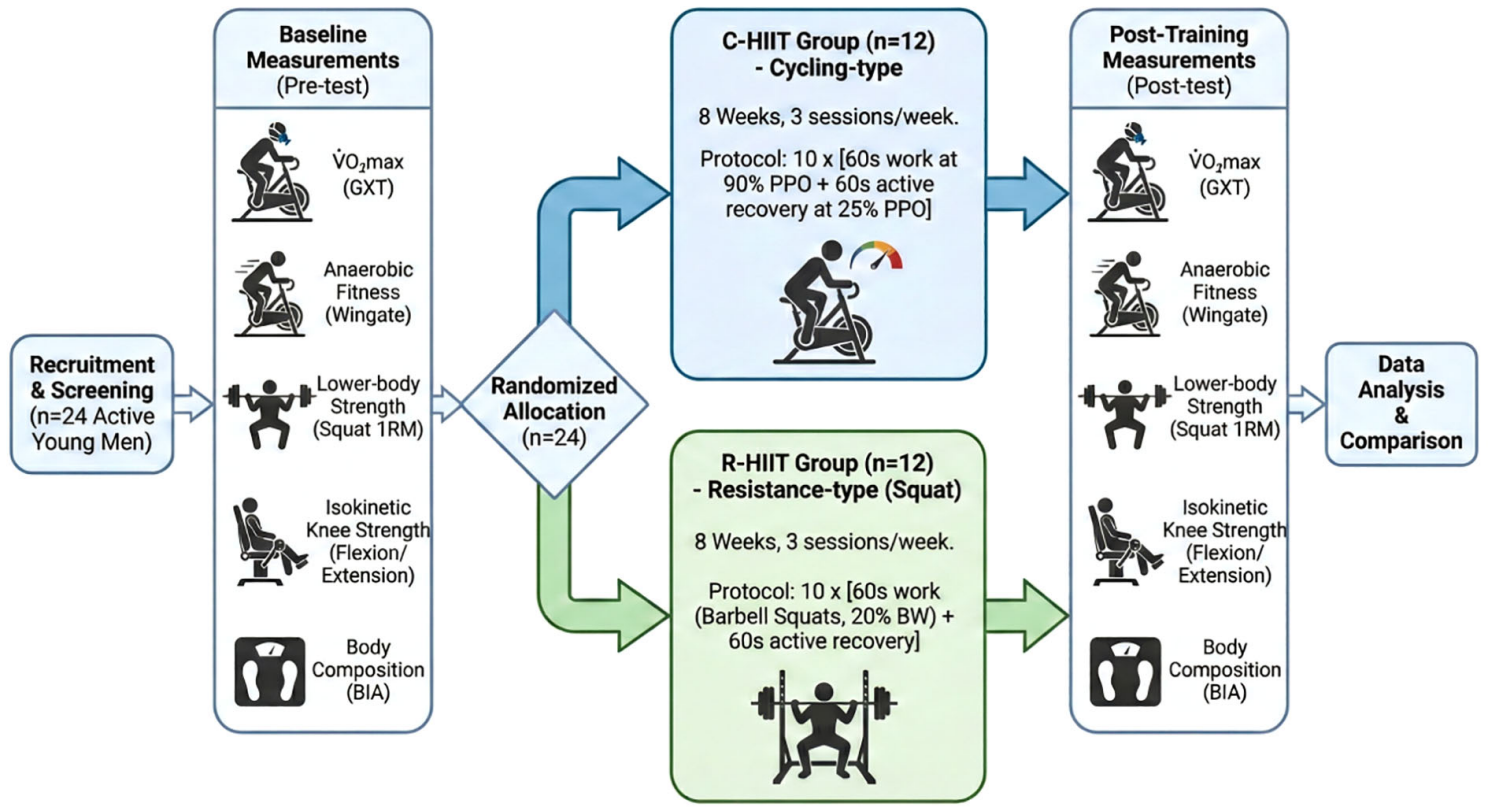
A schematic representation of the study.

This study employed a two-group, pretest–posttest randomized controlled design. Eligible participants were randomly assigned to either a cycling-type high-intensity interval training (C-HIIT) group or a resistance-type high-intensity interval training (R-HIIT) group. Both groups completed an 8-week supervised training intervention (three sessions per week). Primary outcomes included cardiorespiratory fitness (V̇O_2max_), anaerobic fitness (30s Wingate test), lower-body maximal strength (back squat 1RM), isokinetic knee strength, and body composition. All assessments were conducted by blinded assessors before and after the intervention (**Figure 1**).

### Participants

Using G*Power (version 3.1.9.2, Germany) to pre-calculate the sample size, referencing previous similar research designs, the Type I error rate (α) was set at 0.05, with test power (1-β) set at 0.8, and effect size set at 0.4. Using repeated measures analysis for between-group and within-group data with two groups, the minimum sample size for this experiment was calculated as 16 participants, requiring a minimum of 8 participants per group. A cohort of 26 healthy and physically active male volunteers was recruited from the Capital University of Physical Education and Sports in Beijing, China. During the trial, two participants (one from each group) dropped out for reasons unrelated to the intervention. The remaining subjects were randomly allocated into two groups: cycling-type high-intensity interval training (C-HIIT) group (n=12) and a resistance-type high-intensity interval training (R-HIIT) group (n=12). The means and standard deviations (SDs) of age, body height, and body mass were 19.81 ± 1.11 years, 181.50 ± 5.82 cm, and 72.87 ± 8.52 kg for the R-HIIT group, and 19.64 ± 0.81 years, 180.82 ± 3.81 cm, and 71.91 ± 12.21 kg for the C-HIIT group, respectively (**Table 1**). All participants were habitually active but had not been engaged in any structured exercise program (defined as ≥30 min/day, ≥2 days/week) for at least 1 year prior to the study. Prior to the study, visits to the laboratory were conducted, where written informed consent was obtained following a full explanation of the aims, methods, and potential risks. The research adhered to the Declaration of Helsinki and ethical clearance was obtained from the Capital University of Physical Education and Sports Ethical Committee. Additionally, this CONSORT-compliant randomized controlled trial was registered on the Chinese Clinical Trial Registry (www.chictr.org.cn) under the identifier ChiCTR2200056897 on 22/02/2022. Participants were recruited for this study from 13/05/2024 to 20/10/2024.

**Table 1:** Age and physical characteristics before training in both groups (mean and SD).

Variable	R-HIIT (n=12)	C-HIIT (n=12)
Age (year)	19.83 ± 1.11	19.64 ± 0.81
Height (cm)	181.50 ± 5.82	180.82 ± 3.81
Weight (kg)	72.87 ± 8.52	71.91 ± 12.21

### Measurements

#### Anthropometrics

Body height was measured using a wall-mounted stadiometer, and body mass was assessed using a calibrated digital scale (DHM-200, China). Measurements were taken with participants wearing light clothing and no shoes.

### Cardiorespiratory fitness (V̇O_2max_)

We measured V̇O_2max_ using a graded exercise test (GXT) administered on a cycling ergometer (ergoline 100K, Germany). Our protocol started with a 5-min warm-up at 50W. We then initiated the incremental phase at 70W, ramping up the load by 15W/min. The test was terminated when a participant reached voluntary exhaustion, defined as an inability to maintain 60 rpm for over 5 s in a row despite verbal prompting. We collected and analyzed expired gases throughout the GXT with an AEI moxus gas analyzer (AEI moxus, United States) (Wang et al., 2024). If participants were unable to sustain the prescribed workload for the entire 60-s interval, peak power output (PPO) was determined based on the workload attained at volitional exhaustion. PPO was calculated using the following equation: PPO = W_final_ + (t/T) × W_inc_, where W_final_ represents the highest workload completed for a full 60 s. t denotes the time achieved in the final incomplete stage; T is the duration of each stage (60 s); and W_inc_ indicates the incremental increase in workload (15W).

### Wingate anaerobic test

Anaerobic performance was evaluated using a 30-s Wingate test performed on a Monark 894E cycle ergometer (Sweden). The applied resistance was set at 7.5% of the participant’s body mass. During the test, peak power (PP) and mean power (MP) were recorded. Prior to the test, participants completed a standardized 5-min warm-up, consisting of 3 min of low-intensity cycling followed by 2 min of dynamic stretching. At the end of each minute during the warm-up, a 5-s all-out sprint was performed to familiarize participants with the maximal effort required (Song and Sheykhlouvand, 2024).

### Lower-body maximal strength (back squat 1RM)

The one-repetition maximum (1RM) of the back squat was assessed using a barbell and plates on a power rack. Participants refrained from exercise for at least 48 h prior to testing. The protocol followed the NSCA guidelines and was supervised by a certified strength and conditioning specialist. Before the formal 1RM test, participants completed familiarization trials to ensure correct squat technique and to determine an appropriate starting load. The initial warm-up loads were conservatively selected by the certified strength and conditioning specialist based on each participant’s body mass, training experience, movement technique, and perceived exertion during the familiarization trials. After a general warm-up consisting of 5 min of jogging and approximately 10 min of dynamic stretching for the lower body, participants performed a specific warm-up consisting of 8–10 repetitions with a light-to-moderate load, followed by two sets of 2–3 repetitions with progressively heavier loads. Thereafter, single-repetition attempts were performed with progressively increased loads until the 1RM was determined. Each attempt was separated by 3–5 min of rest, and the 1RM was established within five attempts (Mao et al., 2023).

### Isokinetic knee strength

Isokinetic strength of the knee extensors and flexors was evaluated using an Isomed 2000 dynamometer (Germany). Participants were positioned upright in the dynamometer chair, with straps securing the trunk and right thigh to minimize extraneous movement. The right leg was connected to the dynamometer arm, ensuring its rotational axis was aligned with the knee joint axis using the lateral femoral condyle as an anatomical reference.

Assessment involved concentric knee extension and flexion at an angular velocity of 60°·s^-1^. Testing at each velocity started with three submaximal followed by two to three maximal repetitions for warm-up purposes. Three maximal repetitions were then performed at 60°· s^-1^. Once the three maximal repetitions had been tested, each subject was given a brief period of volitional recovery (approximately 5 min). The peak torque (in N·m) was recorded. Throughout the trials, each participant received verbal encouragement to produce maximal efforts during the actual test phase (Pincivero et al., 1997).

### Body composition

Body composition, including fat mass (FM), muscle mass (MM), and lean body mass (LBM), was evaluated using bioelectrical impedance analysis (Inbody 270, Korea). The assessment was performed under strict, standardized protocols. Participants, who were overnight-fasted, had refrained from water, and had an empty bladder, were tested in the early morning while wearing light clothing and no footwear. After entering the participant’s age, sex, and height into the software, body mass was first measured as they stood barefoot on the device. This was immediately followed by the participant grasping the handles with arms extended alongside the body to complete the body-composition assessment, which was completed in less than 30 s. In the present study, total fat mass, muscle mass, and lean body mass were included in the outcome analysis (Mao et al., 2023).

### Training intervention

The C-HIIT and R-HIIT protocols were continuously performed three times a week for 8 weeks. All training sessions for both groups were directly supervised by a research assistant and total duration of training was matched. Training began at least 7 days after the first baseline testing visit and consisted of 24 training sessions over the course of 8 weeks. There was 24–72 h of recovery between training sessions. Previous studies from our laboratory have demonstrated that both the C-HIIT and R-HIIT protocols elicit similar heart rate responses and ratings of perceived exertion (Mao et al., 2022). This method of using multidimensional convergent indicators (e.g., heart rate and perceived exertion) to match the intensity of different training modalities has also been adopted by other researchers (Järvinen et al., 2022; Rønnestad et al., 2020).

For the C-HIIT group, participants completed a 10-min warm-up prior to the training program, including dynamic stretches and 3 min of cycling at 50W. Following a 1-min transition period, the official training began. The C-HIIT protocol, was performed on a cycle ergometer (ergoline 100K, Germany), and comprised ten 60-s work intervals at 90% PPO; each interval was followed by a 60-s active recovery at 25% PPO. Participants were instructed to maintain a pedaling cadence between 60 and 65 rpm throughout the training (**Figure 1**).

For the R-HIIT group, the warm-up lasted approximately 10 min and included dynamic stretching and unloaded squats. The training consisted of 10 × 60-s barbell back squat, interspersed with a 60-s active recovery. During work intervals, participants performed barbell back squats with a load of 20% bodyweight. To ensure correct technique, researchers assisted participants in positioning the barbell while keeping both hands on the bar. They were instructed to squat down to approximately 90° knee flexion with feet flat on the floor and to achieve full hip and knee extension at the end of each repetition. The eccentric phase of each squat lasted 1 s, while the concentric phase was performed as rapidly as possible back to standing position. A maximum of 30 squats were permitted per work interval. Movement tempo was regulated using a metronome set at 60 beats/min. If participants could not maintain the prescribed tempo, they were allowed to briefly pause while in standing position with the load. Between intervals, the researcher removed the barbell and replaced it on the participant’s trapezius 5 s before the next interval began (**Figure 1**).

### Statistical analyses

All statistical analyses were performed using Excel 2019 and SPSS (Version 24.0). Pre- and post-intervention results were presented as means with standard deviations (SD). For primary outcomes, a two-by-two repeated-measures ANOVA (time × group) was applied, followed by *post-hoc* tests with Bonferroni correction for multiple comparisons. Effect sizes were interpreted as follows: for *η_p_^2^*, values of 0.01, 0.06, and 0.14 were considered small, medium, and large, respectively.

## Results

### Cardiorespiratory fitness (V̇O_2max_)

CRF is expressed relative to body weight, with V̇O_2max_ expressed as mL/kg/min accounting for different body size. The V̇O_2max_ values for the two C-HIIT trials are given in **Table 2**, with no significant time × group interaction effect (F = 1.650, P>0.05, *η_p_^2^* = 0.070) and group main effect (F = 0.262, P>0.05, *η_p_^2^* = 0.012). However, the time main effect was significant (F = 16.215, P = 0.001, *η_p_^2^* = 0.424).

**Table 2:** Changes in cardiorespiratory fitness following 8 weeks of training.

Variable	R-HIIT (n=12)		C-HIIT (n=12)		ANOVA		
	Pre	Post	Pre	Post	Group	Time	Interaction
V̇O_2max_ (mL/min/kg)	44.68 ± 5.58	46.79 ± 6.34	42.52 ± 5.91	46.60 ± 5.83	0.614	0.001	0.212
V̇O_2max_ (L/min)	3.24 ± 0.50	3.37 ± 0.51	3.12 ± 0.48	3.37 ± 0.56	0.781	0.001	0.203

### Anaerobic fitness (Wingate cycle test)

There was no significant time × group interaction effect (F = 1.276, P = 0.271, *η_p_^2^* = 0.055) and group main effect (F = 0.449, P = 0.510, *η_p_^2^* = 0.020) for average power output, but there was a significant time main effect (F = 26.50, P<0.001, *η_p_^2^* = 0.546) observed for average power output (**Table 3**). There was no significant time × group interaction effect (F = 0.075, P>0.05, *η_p_^2^* = 0.003) for peak power output, but there was a significant time main effect (F = 25.455, P<0.001, *η_p_^2^* = 0.536) (**Table 3**).

**Table 3:** Changes in anaerobic fitness following 8 weeks of training.

Variable	R-HIIT (n=12)		C-HIIT (n=12)		ANOVA		
	Pre	Post	Pre	Post	Group	Time	Interaction
Average power output (W)	394.75 ± 91.23	473.00 ± 157.62	402.83 ± 88.88	525.08 ± 128.05	0.510	<0.001	0.271
Peak power output (W)	615.25 ± 115.61	761.67 ± 150.69	555.33 ± 106.77	686.67 ± 206.60	0.231	<0.001	0.787

### Back squat 1RM

Data for the measurements of lower-body muscle maximal strength are presented in **Table 4**. There was a significant time main effect (F = 21.757, P<0.001, *η_p_^2^* = 0.497) and time × group interaction effect (F = 7.035, P = 0.015, *η_p_^2^* = 0.242) for back squat 1RM. The pairwise comparisons derived from *post-hoc* analysis show a significant increase in the back squat 1RM between the pretest and the posttest only in the R-HIIT group (P<0.001). Furthermore, after the 8-week intervention, the R-HIIT group demonstrated a greater improvement in back squat 1RM compared to the C-HIIT group (P = 0.048).

**Table 4:** Changes in lower-body strength following 8 weeks of training.

Variable	R-HIIT (n=12)		C-HIIT (n=12)		ANOVA		
	Pre	Post	Pre	Post	Group	Time	Interaction
Back squat 1RM (kg)	82.08 ± 22.81	98.75 ± 14.64***#	80.83 ± 15.64	85.42 ± 16.44	0.298	<0.001	0.015
Knee flexion peak torque at 60°· s^-1^ (N·m)	85.92 ± 40.01	141.42 ± 40.20***#	93.33 ± 22.78	109.25 ± 27.06*	0.353	<0.001	<0.001
Knee extension peak torque at 60°· s^-1^ (N·m)	171.75 ± 35.78	197.00 ± 42.25	177.25 ± 63.27	207.75 ± 60.66	0.696	<0.001	0.614

Values are presented as mean ± SD. *P < 0.05, ***P < 0.001 vs. pre-intervention within the same group; #P < 0.05 vs. C-HIIT at post-intervention. P values for group, time, and group × time interaction effects were obtained from two-way repeated-measures ANOVA.

### Knee flexion and extension

There was a significant interaction effect of time × group knee flexion peak torque at 60°·s^-1^ (F = 23.811, P<0.001, *η_p_^2^* = 0.520). The main effect of time was significant for knee flexion peak torque at 60°·s^-1^ (F = 77.510, P<0.001, *η_p_^2^* = 0.779). *Post-hoc* analysis revealed that both the R-HIIT group (P<0.001) and the C-HIIT group (P = 0.011) showed a significant increase in knee flexion peak torque at 60°·s^-^¹. Furthermore, after the 8-week intervention, the R-HIIT group demonstrated a greater improvement in knee flexion peak torque at 60°·s^-1^ compared to the C-HIIT group (P = 0.031) (**Table 4**).

There was no interaction effect of time × group knee extension peak torque at 60°·s^-1^ (F = 0.262, P = 0.614, *η_p_^2^* = 0.012). The main effect of time was significant for knee extension peak torque at 60°·s^-1^ (F = 29.534, P<0.001, *η_p_^2^* = 0.573) (**Table 4**).

### Body composition

As shown in **Table 5**, muscle mass (MM) and lean body mass (LBM) were unaffected by the 8-week training period for both groups. However, fat mass (FM) was affected by time both in the C-HIIT and R-HIIT groups (F = 11.104, P<0.05, *η_p_^2^* = 0.335).

**Table 5:** Change in the body composition following 8 weeks of training.

Variable	R-HIIT (n=12)		C-HIIT (n=12)		ANOVA		
	Pre	Post	Pre	Post	Group	Time	Interaction
Body weight (kg)	72.87 ± 8.52	72.17 ± 7.76	71.51 ± 11.83	70.59 ± 11.91	0.727	0.024	0.748
Fat mass (kg)	9.31 ± 2.06	8.68 ± 1.99	11.58 ± 4.75	10.75 ± 4.69	0.155	0.003	0.681
Muscle mass (kg)	35.97 ± 5.08	35.96 ± 4.99	33.95 ± 4.68	34.08 ± 4.51	0.331	0.687	0.645
Lean body mass (kg)	63.56 ± 8.39	63.48 ± 8.22	59.93 ± 7.79	59.84 ± 7.64	0.297	0.777	0.950

## Discussion

The major finding was that there were significant improvements in the effects on cardiorespiratory fitness, anaerobic fitness, knee flexion and extension peak torque, and body composition both C-HIIT and R-HIIT. However, we found that R-HIIT was more effective than C-HIIT in back squat 1RM and knee flexion peak torque.

Both C-HIIT and R-HIIT significantly improved V̇O_2max_, with no significant time × group interaction. Therefore, the present findings indicate that both protocols produced comparable improvements in cardiorespiratory fitness. These improvements may be related to the high-intensity interval structure shared by both protocols (Marterer et al., 2020), the matched internal training load, and repeated exposure to short bouts of vigorous lower-limb exercise. Therefore, R-HIIT may provide a sufficient cardiorespiratory stimulus despite being organized around resistance-type movements. An improvement in cardiorespiratory fitness was observed following 8 weeks of R-HIIT in active healthy men, supporting the time-efficient nature of this approach. The participants mainly performed deep squat exercises with limited rest intervals, yet still achieved significant gains in cardiorespiratory fitness—a finding consistent with earlier circuit-training studies (Myers et al., 2015). Although the participants engaged in only minimal traditional endurance exercise, the short bouts of maximal-intensity training they completed have been previously linked to enhancements in cardiovascular function (Gibala and McGee, 2008; Macpherson et al., 2011). Moreover, these results in humans align with prior animal research indicating that HIIT can improve skeletal muscle oxidative capacity (Dudley et al., 1982). Notably, although R-HIIT involved weighted deep squats as a form of resistance exercise, it followed the typical HIIT structure in terms of timing, and heart rate was used for intensity regulation, leading to cardiorespiratory improvements comparable to those seen with C-HIIT (Douglas, 2017; K. et al., 2002). This is further supported by our earlier acute experiments, which showed that a single session of R-HIIT elicits cardiorespiratory responses similar to C-HIIT (Mao et al., 2022).

Both C-HIIT and R-HIIT significantly increased mean and peak power during the Wingate test, but no significant time × group interaction was observed. These findings suggest that the two protocols produced broadly comparable improvements in anaerobic performance. Although improvements in lower-body strength may contribute to better power production during cycling-based anaerobic tests (Alemdaroğlu, 2012; Rønnestad et al., 2010), the present data do not support a statistically greater anaerobic adaptation for R-HIIT than C-HIIT. In past studies in our laboratory, R-HIIT was found to cause a higher acute response of blood lactate than C-HIIT (Mao et al., 2022). This suggests that R-HIIT is dominated by an anaerobic glycolytic energy supply system. However, there was no statistically significant difference in anaerobic fitness performance between the long-term C-HIIT and R-HIIT interventions. Previous studies have shown that cycling-type HIIT can significantly improve anaerobic fitness, which supports the findings of our study (Buckley et al., 2015; Gist et al., 2014). We can only speculate that the effects of long-term R-HIIT and C-HIIT interventions on changes in anaerobic metabolism are similar. Since this study did not measure glycolytic enzyme activity, muscle buffering capacity, or ion regulation, it is not possible to directly determine the mechanisms underlying anaerobic metabolism; therefore, only speculative interpretations regarding anaerobic metabolic adaptations can be proposed.

For back squat 1RM, the greater improvement observed after R-HIIT may be largely explained by the specificity of the training stimulus. The R-HIIT protocol was based on repeated barbell back squats, which closely matched the movement pattern of the 1RM test. Therefore, improvements in squat performance may have resulted from better movement coordination, increased familiarity with the squat pattern, improved intermuscular coordination, and enhanced neuromuscular efficiency during the task (Akagi et al., 2020). The rapid concentric phase required in each squat repetition may also have provided a repeated stimulus for force production in the lower limbs (Sugisaki et al., 2014). In contrast, cycling-type HIIT provides a strong lower-limb metabolic and cardiovascular stimulus, but it does not reproduce the movement pattern or loading characteristics of the back squat test (Andrade et al., 2023). Importantly, the strength gain observed in the R-HIIT group should not be interpreted primarily as evidence of muscle hypertrophy, because muscle mass and lean body mass did not significantly increase after the intervention. Thus, the improvement in 1RM is more likely to reflect functional and neuromuscular adaptations rather than measurable increases in muscle mass (Myers et al., 2015; Schaun, 2019). This interpretation is consistent with the present body-composition results, in which muscle mass and lean body mass remained unchanged.

For isokinetic knee strength, both protocols improved knee extension peak torque, whereas R-HIIT induced a greater improvement in knee flexion peak torque. Previous squat-training research has shown that squat exercises can stimulate both the knee extensors and flexors (Kubo et al., 2019), which may partly explain the improvement in knee strength observed after the squat-based R-HIIT protocol. Although the external load used in the present R-HIIT protocol was relatively low, the repeated high-intensity squat movements required participants to perform repeated hip and knee extension while maintaining lower-limb stability throughout the movement (Buckley et al., 2015; K. et al., 2002). These repeated neuromuscular demands may have contributed to the improvement in knee flexion and extension peak torque. The greater improvement in knee flexion strength following R-HIIT may be related to the specific role of the knee flexors during the squat movement (Sugisaki et al., 2014). During repeated squatting, the hamstrings contribute not only to hip extension but also to lower-limb stabilization and movement control, particularly when the exercise is performed repeatedly under fatigue (Bolgla et al., 2016; Fry et al., 2003). In contrast, cycling is generally more quadriceps-dominant during the downstroke and may provide a less specific stimulus for knee flexor strength (Martin et al., 1994). Nevertheless, C-HIIT also improved knee strength, which is consistent with previous findings showing that cycling-based interval training can increase isokinetic knee torque (Martin et al., 1994). Therefore, the improvement in knee extension peak torque in both groups may reflect a general lower-limb adaptation to repeated high-intensity exercise, whereas the greater improvement in knee flexion peak torque after R-HIIT may be more closely related to the movement-specific neuromuscular demands of the squat exercise. Because muscle activation patterns and muscle morphology were not directly measured, this explanation should be considered a plausible interpretation rather than a confirmed mechanism.

Regarding body composition, both C-HIIT and R-HIIT reduced fat mass after the 8-week intervention, whereas muscle mass and lean body mass remained unchanged. A previous study (Racil, 2016) showed improved body mass among obese women after a total of 108-min HIIT. The reason could be that C-HIIT and R-HIIT not only cause energy expenditure during exercise, they also lead to negative energy balance and an increase in excess post-exercise oxygen consumption (EPOC) due to their high exercise intensity (Laforgia et al., 1997). Notably, EPOC is usually more affected by exercise intensity (Laforgia et al., 1997). Previous research conducted in our laboratory has demonstrated that there is no difference in the acute heart rate response between C-HIIT and R-HIIT (Mao et al., 2022), so there was no difference in the effect of C-HIIT and R-HIIT on fat mass. Moreover, high-intensity exercise increases the activity of enzymes and proteins involved in beta oxidation (Burgomaster et al., 2005; Rodas and CussoÂ, 2000; Tremblay et al., 1994). However, our study found that fat mass was significantly improved after an 8-week R-HIIT, while lean body mass was unaltered. A previous study showed that high-intensity functional training (HIFT) can increase lean body mass (Heinrich, 2015; Lu et al., 2021; Poston et al., 2016). This may be because the R-HIIT in our study involved only lower-limb muscle groups, whereas HIFT involves whole-body muscle groups. So, R-HIIT was not sufficient to improve lean body mass. Note that lower-limb segmental lean mass was not analyzed in the present study. Therefore, although the R-HIIT protocol was based on a lower-body squat movement, we cannot determine whether regional changes in lower-limb lean mass occurred.

## Conclusions

Eight weeks (three times per week) of C-HIIT and R-HIIT produced comparable improvements in V̇O_2max_, anaerobic fitness, knee extension peak torque, and fat mass. Compared with C-HIIT, R-HIIT induced greater improvements in back squat 1RM and knee flexion peak torque. Neither C-HIIT nor R-HIIT significantly increased lean body mass.

## Limitations

Several limitations should be acknowledged. First, the sample size was relatively small and included only active healthy young men, which may limit the generalizability of the findings to women, sedentary individuals, older adults, or athletic populations. Second, although body composition was assessed using bioelectrical impedance analysis, only total fat mass, muscle mass, and lean body mass were included in the outcome analysis. Lower-limb segmental lean mass was not analyzed; therefore, the present study cannot determine whether regional changes in lower-limb lean mass occurred after the squat-based R-HIIT protocol. Third, the present study mainly focused on changes in performance-related outcomes and body composition, while the physiological mechanisms underlying these adaptations were not directly examined.

## Data Availability

The datasets presented in this study can be found in online repositories. The names of the repository/repositories and accession number(s) can be found in the article/supplementary material.
